# Next-Generation Multi-Engager Complexes Linking Natural Killer Cells to Tumor Cells and Targeting the Proteolytic Checkpoint ADAM17

**DOI:** 10.3390/cells15141269

**Published:** 2026-07-15

**Authors:** Kate J. Dixon, Bruce Walcheck

**Affiliations:** 1 Department of Veterinary and Biomedical Sciences, University of Minnesota, St. Paul, MN 55108, USA; walch003@umn.edu; 2 Masonic Cancer Center, University of Minnesota, Minneapolis, MN 55455, USA; 3 Center for Immunology, University of Minnesota, Minneapolis, MN 55455, USA; 4 Stem Cell Institute, University of Minnesota, Minneapolis, MN 55455, USA

**Keywords:** NK cells, ADAM17, engager complexes, ADCC, immunotherapy, cancer, immunosuppression

## Abstract

Natural killer (NK) cells are innate lymphocytes capable of killing transformed cells without prior sensitization through direct cytolytic activity and, in an antigen-specific manner, by antibody-dependent cellular cytotoxicity (ADCC). Human NK cells mediate ADCC exclusively via the IgG Fc receptor CD16 (FcγRIIIA), triggering degranulation and cytokine production. Multi-engager complexes, such as BiKEs, TriKEs, TetraKEs, ANKETs, and ICEs, have been developed to link CD16 to tumor antigens. However, CD16 expression can be rapidly downregulated upon NK cell activation by the proteolytic checkpoint, a disintegrin and metalloproteinase-17 (ADAM17). ADAM17 induction in NK cells occurs in response to various stimuli, including the potent activating receptor CD16 and cytokine signaling. CD16 downregulation is further exacerbated within the tumor microenvironment, significantly diminishing ADCC potency by tumor-infiltrating NK cells. Moreover, ADAM17 expression and function are upregulated in various solid tumors, leading to the release of NK cell ligands and tumor-promoting factors. Consequently, developing strategies to inhibit ADAM17 to enhance NK cell function and suppress tumor cell growth is paramount to multi-engager efficacy. This review provides an overview of NK cell biology and the role of ADAM17 in regulating NK cell function and tumor cell growth. We examine the current landscape of NK cell multi-engager complexes in clinical development and discuss emerging strategies for incorporating an ADAM17-blocking component to optimize therapeutic outcomes.

## Simple Summary

Natural killer (NK) cells are innate cytolytic lymphocytes that kill tumor cells. Multi-engager complexes are being developed to link CD16 on NK cells to tumor cell antigens, but their efficacy is limited by the proteolytic checkpoint ADAM17, which cleaves CD16. Novel engager complexes that also target ADAM17 are discussed to preserve key substrates, thereby enhancing NK cell function and suppressing tumor cell growth.

## 1. Introduction

Unlike T cells, NK cells recognize stressed, transformed, and virally infected cells without prior antigen sensitization [1]. NK cells mediate cytotoxicity through two mechanisms: natural cytotoxicity and antibody-dependent cellular cytotoxicity (ADCC) [1,2,3]. Natural cytotoxicity is governed by the balance of signals from activating and inhibitory receptors [2,4]. In contrast, ADCC is triggered in human NK cells solely by CD16 (FcγRIIIA) engagement of cell-bound IgG (primarily IgG1), the only NK cell activating receptor capable of inducing full degranulation without co-stimulation [1,5]. Upon activation, NK cells initiate signaling cascades that mobilize cytolytic granules and facilitate the lysis of target cells [2,4]. In addition, activated NK cells produce pro-inflammatory cytokines such as IFN-γ, TNF-α, and chemokines including CCL3, CCL4, and CCL5 [2,4]. These secreted signals modulate additional innate and adaptive immune responses, such as the maturation of antigen-presenting dendritic cells, MHC class I and II expression, T cell recruitment, and polarization of T_H_1 cells [1]. These properties have positioned NK cells as attractive candidates for cancer immunotherapy.

Chimeric antigen receptor (CAR) T cell therapies have transformed the treatment of certain hematologic malignancies [6]; however, their application is constrained by significant risks of cytokine release syndrome (CRS) and immune effector cell-associated neurotoxicity syndrome (ICANS), which occur in the majority of treated patients [7,8]. Additionally, autologous manufacturing and administration of CAR T cells add substantial logistical complexity and cost, and CAR single-antigen targeting creates selection pressure for resistance and antigen-escape relapse [9,10]. Clinical evidence demonstrates that autologous NK cell infusions carry substantially lower risks of CRS and ICANS. Moreover, allogeneic NK cells can also kill tumor cells by missing-self mechanisms and do not cause graft-versus-host disease (GvHD), enabling the development of off-the-shelf products that circumvent the manufacturing burdens of individualized therapy [11,12]. Finally, NK cells can be directed against a broad range of tumor antigens by antibody therapies [5,13].

Clinical interests are evolving from monoclonal antibody (mAbs) therapies toward a more flexible, modular system of NK cell multi-engager complexes that directly link NK cells to tumor cell antigens and incorporate cytokine stimulation [14,15,16]. CD16 binding affinity to IgG varies due to genetic polymorphisms, whereas NK cell engager molecules directly bind to CD16 and therefore their induction of signaling is less constrained by CD16 allelic variants [14]. This review provides an overview of NK cell biology, the function and regulation of ADAM17 in NK cells as well as tumor cells, and the current landscape of NK cell multi-engager complexes, with a focus on next-generation versions that incorporate an anti-ADAM17 component for blocking and targeting in NK and tumor cells.

## 2. NK Cell Biology

### 2.1. NK Cell Subsets and Maturation

NK cells are innate immune cells of the CD3^−^CD56^+^ lineage in humans that develop from common lymphoid progenitors [1,4]. NK cells primarily develop and mature in the bone marrow, though they also mature in secondary lymphoid organs [1,4]. Human NK cells are conventionally divided into two major subsets defined by differential expression of CD56 and CD16: the CD56^bright^ (CD16^dim/−^) subset and the CD56^dim^ (CD16^+^) subset [1]. CD56^bright^ NK cells represent approximately 5–15% of circulating NK cells and are enriched in secondary lymphoid organs, particularly lymph nodes and tonsils [1]. These cells are less terminally differentiated, express high levels of CD94/NKG2A inhibitory complexes, and characteristically produce abundant pro-inflammatory cytokines, particularly IFN-γ and TNF-α, in response to cytokine stimulation [2]. CD56^bright^ NK cells express low or no surface CD16 and therefore contribute minimally to ADCC [2,17]; however, these cells retain developmental plasticity [17]. Upon appropriate cytokine stimulation, particularly with IL-2 and IL-15, they can differentiate into the CD56^dim^ phenotype [4]. CD56^dim^ NK cells are the major circulating subset, comprising approximately 85–95% of peripheral blood NK cells [1]. These terminally differentiated cells are characterized by high CD16 expression, cytolytic granules containing perforin and granzymes, and cytotoxic capability against tumor and virally infected targets [1]. The CD56^dim^ subset is the primary effector subset that mediates natural cytotoxicity and ADCC [1,3].

### 2.2. NK Cell Induction of Cytotoxicity

Natural cytotoxicity is triggered when activating receptor signals exceed inhibitory thresholds established by killer cell immunoglobulin-like receptors (KIRs) and CD94/NKG2A heterodimers, which recognize classic MHC class I molecules and the non-classic HLA-E molecule, respectively [2]. The ‘missing self’ principle describes the ability of NK cells to lyse targets with reduced, absent, or different MHC class I expression [18]. Ligands for activating receptors, such as NKG2D, NKp30, NKp46, and DNAM-1, are frequently upregulated on malignant and stressed cells, providing additional stimulatory signals to counteract tumor cell inhibitory signaling [3,19,20]. ADCC by human NK cells is mediated exclusively by CD16, which recognizes the Fc region of IgG antibodies bound to cell-surface antigens on target cells [21]. Because CD16-mediated signaling is uniquely potent, its activation threshold can override dominant inhibitory KIR signals, driving full degranulation even against HLA-sufficient tumors [3,22]. CD16-mediated ADCC represents the primary mechanism of tumor killing for several FDA-approved therapeutic antibodies [23,24,25].

Upon NK cell activation, cytolytic effector mechanisms are initiated through two principal pathways. The granule exocytosis pathway is the dominant mechanism for direct target-cell killing [2]. Cytolytic granules containing perforin and serine proteases (granzymes) polarize toward the immunological synapse via microtubule-organizing center (MTOC) reorientation [2]. Perforin polymerizes within the target cell membrane to form transmembrane pores, facilitating the entry of granzymes A and B [26]. The latter directly cleaves and activates caspase-3, initiating intrinsic apoptosis and mitochondrial cytochrome c release [26]. Granzyme A induces caspase-independent single-stranded DNA damage [26]. A second cytotoxic pathway operates through death receptor ligands. NK cells constitutively express TRAIL and upregulate FasL, which engage DR4/DR5 and Fas, respectively, on susceptible tumor cells to trigger extrinsic apoptosis [2,26]. The relative contribution of each pathway varies with target cell phenotype and activation context, but together they ensure rapid and decisive target cell elimination.

CD16 lacks an intrinsic signaling capacity and instead associates non-covalently with the CD3ζ chain and the FcRγ chain, which form homo- and heterodimers bearing immunoreceptor tyrosine-based activation motifs (ITAMs) in their cytoplasmic tails [2,3]. Engagement of CD16 by IgG-opsonized target cells induces receptor clustering and ITAM phosphorylation by Src-family kinases, including Lck and Fyn [2,13]. Phosphorylated ITAMs serve as docking sites for the protein tyrosine kinases Syk and ZAP-70, which initiate downstream signaling cascades [2]. This includes activation of PLCγ, which generates IP3 and DAG, triggering calcium flux and PKC activation [2]. Parallel activation of the PI3K/Akt and MAP kinase pathways completes the signaling response, collectively driving polarization of cytolytic granules toward the immunological synapse, degranulation of perforin and granzymes, and production of IFN-γ, TNF-α, and other cytokines and chemokines [2].

Genetic polymorphisms affect the affinity with which CD16 binds to IgG [27,28,29,30]. The most clinically significant polymorphism occurs at amino acid position 158, arising from a G-to-T point mutation at nucleotide 559 that produces a valine (V) to phenylalanine (F) substitution [27,30]. CD16 158V binds IgG1 with approximately two-fold higher affinity than 158F, yet the latter allele predominates globally, with the F/F homozygous genotype present in ~25% of individuals [29]. The clinical consequences of this polymorphism are well documented. Patients with non-Hodgkin lymphoma treated with rituximab show significantly improved responses when homozygous for 158V, and similar genotype-dependent differences in response rates have been reported for cetuximab and trastuzumab [31,32,33].

Unlike other activating receptors on NK cells, CD16 surface expression is post-translationally regulated by ADAM17 [34,35,36]. ADAM17 efficiently cleaves cell surface CD16, which is a rapidly induced (within minutes) event that can result in a significant loss of cell-surface CD16 [34,35,36,37]. ADAM17 activity is induced by CD16 during ADCC, other activating receptors, various cytokines, and in the tumor microenvironment [37,38]. This process likely functions as a negative feedback mechanism that regulates the immunological synapse and effector-target conjugation [37]. Srpan et al. reported that blocking ADAM17 cleavage of CD16 decreased the detachment of NK cells from target cells, reducing sequential killing [39]. This effect, however, has not been demonstrated in vivo and how this impacts sequential killing in a tumor microenvironment has not been elucidated. However, because evolutionary pressure rarely preserves a rapid, drastic receptor loss without functional benefit, this mechanism likely evolved to optimize responses and/or mitigate excessive inflammation during infectious diseases rather than malignancy, given that the evolutionary burden of cancer predominantly falls after reproductive age. Indeed, CD16 shedding appears to be detrimental to the anti-tumor function of NK cells [37,40]. This process can be blocked by various approaches, including site-directed mutagenesis of CD16, mAbs directed to its cleavage region, small-molecule inhibitors of ADAM17, and mAbs directed to ADAM17 [34,41,42,43,44]. Notably, preventing CD16 shedding has been shown in multiple studies to enhance ADCC and IFN-γ production by NK cells both in vitro and in vivo [43,45,46,47,48].

## 3. ADAM17: Function and Regulation

### 3.1. ADAM17: Discovery and Activation

ADAM17 is a type I transmembrane zinc-dependent metalloprotease that belongs to the adamalysin subfamily of the metzincin metalloproteinase superfamily [49]. ADAM17 is constitutively expressed on the surface of most cell lineages [50]. Studies have demonstrated that ADAM17 processes a broad array of substrates, as reported in other reviews [50,51,52]. A recent study found that ADAM17 had over 100 substrates when evaluated in fibroblasts [53]. The spatial restriction of ADAM17 activity is primarily *cis*, acting on substrates on the same cell surface and typically cleaving them proximal to the cell membrane, a process referred to as ectodomain shedding [54]. The essential physiological role of ADAM17 is underscored by the lethal consequences of its complete absence. Global *Adam17* gene knockout in mice results in perinatal death due to defects in cardiac valvulogenesis, skin barrier malformation, and branching morphogenesis of multiple organ systems, phenotypes that overlap with those of mice lacking EGFR or its ligands, which are substrates of ADAM17, reflecting its critical role in growth factor signaling [55]. In humans, homozygous loss-of-function mutations in ADAM17 cause a severe multisystem disorder characterized by neonatal inflammatory skin and bowel disease, with features including perioral inflammatory dermatitis, bloody diarrhea, and susceptibility to bacterial infection [56]. Due to the ubiquitous expression and broad substrate repertoire, ADAM17 is not dispensable, and blocking its activity systemically for an extended period may pose significant risks, supporting approaches to target specific substrates or to inhibit ADAM17 in a cell-selective manner.

ADAM17 is constitutively expressed and exists in a basal, low-activity state. To traffic to the cell membrane, ADAM17 requires the inactive rhomboid chaperone proteins iRhom1 or iRhom2 for exit from the endoplasmic reticulum [57,58,59]. Notably, while iRhom1 is widely expressed, iRhom2 expression is strictly restricted to hematopoietic cells, dictating leukocyte-specific ADAM17 maturation [58,59]. These iRhoms are essential for the transport of ADAM17 to the Golgi apparatus, where the inhibitory pro-domain of the sheddase is removed by furin-type proprotein convertases, a critical process that renders the enzyme “mature” yet still catalytically inert [58,59,60].

ADAM17 proteolytic activity is highly inducible by cellular activation or stress [54,61,62]. The rate and efficiency of this induction are exemplified in neutrophils, which express 50,000–100,000 surface CD62L (L-selectin) molecules and essentially all are cleaved from the cell surface within minutes upon cell activation [54,63,64]. The induction of ADAM17 activity involves a rapid and reversible conformational shift between “closed” (inactive) and “open” (active) states. In the inactive state, the catalytic site is sterically hindered and inaccessible to substrates [50]. Activation is driven by heterogeneous intracellular signaling pathways that vary by cell type and stimulus. These pathways primarily involve serine and threonine kinases, including MAPKs and PKC [37]. The precise proximal targets of these kinases remain under investigation. In resting cells, ADAM17 may form membrane dimers that associate with the endogenous inhibitor tissue inhibitor of metalloproteinase 3 (TIMP3), which noncovalently blocks the catalytic region [65]. Upon cell activation and MAPK signaling, these inactive dimers convert to active monomers, driving the dissociation of TIMP3 and derepressing the enzyme [65]. ADAM17 conformation has also been reported to be modulated via its disintegrin-like and cysteine-rich domains. These domains contain highly conserved cysteine-X-X-cysteine (CXXC) motifs that function as allosteric disulfide bonds and rapid conformational switches sensitive to reducing/oxidation agents [66]. Together, these regulatory processes ensure that the induction of ADAM17 activity is precisely timed and measured to the cellular response.

### 3.2. ADAM17 Activity in the Tumor Microenvironment

ADAM17 has been shown to be upregulated in numerous malignancies and is associated with a worse prognosis [38,67]. Increased ADAM17 expression has been reported in ovarian cancer, colon cancer, lung cancer, breast cancer, pancreatic cancer, and prostate cancer [52,68,69,70,71,72,73,74]. In ovarian cancer, ADAM17 has been proposed as an early detection biomarker [75]. In breast cancer, specifically triple-negative, ADAM17 mRNA and protein levels are significantly elevated compared to normal breast tissue, and high expression is a strong indicator of shorter overall survival [72]. The correlation with malignancy and poor survival underscores the role that ADAM17-mediated proteolytic activity plays in orchestrating key tumorigenic processes.

ADAM17 is the primary protease responsible for the cleavage of the EGFR ligands transforming growth factor α (TGF-α), amphiregulin, epiregulin, and HB-EGF [38,67]. These soluble factors bind to EGFR on the same tumor cell or on nearby cells and trigger downstream pathways that drive cell proliferation, survival, and metastatic potential [76]. ADAM17-mediated ligand shedding is a mechanism of clinical resistance to EGFR inhibitors. High levels of TGF-α and amphiregulin can outcompete mAbs like cetuximab for receptor binding, leading to poor clinical outcomes [77,78]. ADAM17-mediated cleavage of tumor cell adhesion molecules remodels the cell surface to promote metastasis. For example, the proteolytic shedding of CD44 by ADAM17 drives cancer stemness in head and neck squamous cell carcinomas [79], and is significantly correlated with nodal metastasis and tumor recurrence in oral squamous cell carcinoma patients [80]. Beyond phenotypic remodeling, this protease shapes a tolerogenic tumor microenvironment. For instance, MICA/B and NR3LG1 (also known as B7-H6), stress-inducible ligands for the NK cell activating receptors NKG2D and NKp30, respectively, are shed from tumor cell surfaces by ADAM17 [38,81,82]. Shed MICA/B and NR3LG1 also function as decoy molecules that competitively inhibit NKG2D and NKp30 signaling, contributing to NK cell exhaustion in the tumor microenvironment [40,82,83]. Continuous exposure to these soluble ligands induces endocytosis and lysosomal degradation of NKG2D, resulting in desensitized NK cells [83]. However, blocking the shedding of these ligands has been shown to increase NK cell-mediated tumor killing [82,83].

ADAM17 activation and upregulation are induced by key components of the tumor microenvironment that promote immunosuppression, including TGF-β and hypoxia [61,84]. Hypoxia has been shown to induce ADAM17 expression and activity through multiple mechanisms, including unfolded protein response activation (caused by misfolded proteins), reactive oxygen species, endoplasmic reticulum stress, and stabilization of hypoxia-inducible factor 1-alpha (HIF-1α) [38,61,85,86]. This transcriptional regulator directly binds to the ADAM17 promoter to upregulate its expression [87], whereas HIF-1α silencing prevents ADAM17 upregulation [88,89]. Reactive oxygen species are abundant in the tumor microenvironment due to myeloid-derived suppressor cells and heightened tumor metabolism [90], which have been shown to activate ADAM17 [38]. Together, these converging signals create a tumor microenvironment milieu that strongly favors ADAM17 induction and upregulation, leading to robust substrate downregulation by immune and tumor cells.

### 3.3. CD16 Shedding in NK Cells

We and others have shown that ADAM17 is the primary sheddase of CD16, which is cleaved at a specific membrane-proximal extracellular site, between Ala195 and Val196 [34,35]. An adjacent residue, Ser197, plays a critical role as its substitution with proline (S197P) introduces conformational changes in the juxtamembrane region of CD16 that render the receptor resistant to ADAM17 cleavage [34]. This non-cleavable CD16 variant retains adhesion and signaling function and has been combined with the 158V allele to create a higher-affinity, non-cleavable version of CD16 [47,91,92,93], and is currently being examined in iPSC-derived NK cells and T cells for cancer and autoimmune therapies (NCT04551885, NCT04245722, and NCT05950334).

In solid tumors where ADAM17 activity is augmented, CD16^−^ NK cells have been documented in the tumor infiltrates across multiple malignancies, including prostate, ovarian, and colorectal cancers [94,95,96,97,98]. While high densities of NK cell tumor infiltration are generally associated with favorable patient prognosis, this clinical benefit is contingent upon functional NK cell status. CD16 loss renders tumor-infiltrating NK cells less responsive to antibody-based therapies and CD16-dependent engagers [96,99], diminishing their therapeutic potential. Additionally, ADAM17 cleaves CD62L, which governs NK cell homing to lymphoid organs and proliferative niches [100].

The observation that tumor-infiltrating NK cells exhibit reduced surface CD16, together with ADAM17 induction and upregulation occurring by multiple signals from the tumor microenvironment, suggests that CD16 utilization by antibody-based therapies is a significant barrier in the solid tumor setting. Consequently, the strategic preservation of CD16 is essential to maximize the efficacy of multi-engager complexes that utilize CD16 to mediate cytotoxicity.

## 4. The NK Cell Engager Landscape

### 4.1. Conceptual Architecture: NK Cell Engagers

The evolution of NK cell engager molecules reflects advances in understanding both NK cell biology and the limitations of earlier approaches. The common architectural principle shared by all NK cell engagers is high-avidity bridging of an NK cell-activating receptor (most commonly CD16) with a tumor-associated antigen, thereby directing NK cell cytotoxicity to tumor cells in a manner analogous to how therapeutic IgG antibodies direct ADCC [14,15,16]. Engager molecules, however, link NK cells directly to tumor targets without Fc receptor competition by other antibodies and variability in IgG binding affinity [14,15]. Moreover, they offer multi-specific antigen recognition, tunable affinity, and cytokine moiety additions [14]. Because CD16 is expressed by all mature peripheral blood NK cells, is the only activation receptor capable of independently driving the full effector program, and is not expressed by T or B cells, it is the preferred anchoring receptor for NK cell engager molecules [15]. This modular flexibility has led to the development of several distinct versions of engager architectures.

**Figure 1:**
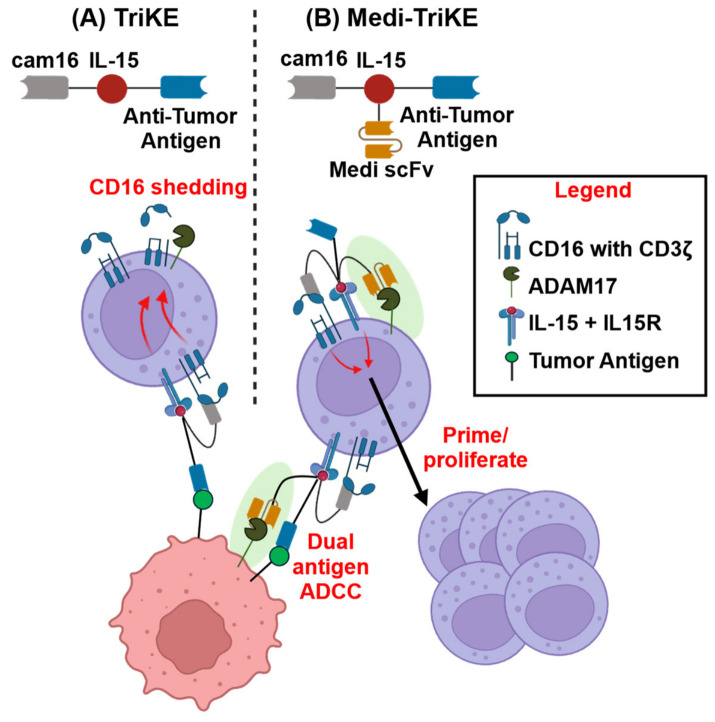
(**A**). TriKE: Anti-CD16 VHH/IL-15/anti-tumor antigen scFv or VHH mediates NK cell linkage to a tumor cell. (**B**). Medi-TriKE: Medi-scFv attached to a TriKE. The added functionality of Medi-scFv is highlighted in green, which includes blocking ADAM17 function in NK cells and targeting ADAM17 in tumor cells.

Bi-specific killer engagers (BiKEs) and tri-specific killer engagers (TriKEs): A BiKE consists of single-chain variable fragments (scFvs) and/or variable domains of a heavy chain-only antibody (VHH) targeting CD16 and a tumor antigen [101]. Early BiKE platforms demonstrated proof-of-concept of NK cell-directed killing but were limited by short serum half-lives, lack of cytokine stimulation, and single-antigen targeting which was vulnerable to antigen escape [102]. A TriKE has the same effector and targeting arms and also consists of an IL-15 moiety as a third component [101,103] (Figure 1). IL-15 is a potent NK cell survival and proliferation cytokine [104]. Current antigen targets with the TriKE platform include B7-H3, CD19, CD33, HER2, and EGFR [101,103,105,106,107,108].

An innate cell engager (ICE) consists of tetravalent bispecific antibody-based platforms developed by Affimed GmbH that use a 2 + 2 format, two anti-CD16 domains and two anti-tumor antigen domains, arranged in a tandem diabody structure [109,110]. The bivalent engagement of both CD16 and the tumor antigen provides higher binding avidity and more potent NK cell activation than monovalent formats [109]. Tumor antigens targeted with the ICE platform include EGFR, BCMA, and CD123 [111,112,113].

Other strategies to target NK cells include adding a second effector arm to an NK cell activation receptor. Innate Pharma developed an antibody-based NK cell engager therapeutic (ANKET) targeting CD16 and NKp46 with a modified IL-2 variant for NK cell proliferation [114]. Dragonfly Therapeutics designed a tri-specific NK cell engager therapy (TriNKET) aiming to target CD16 and NKG2D on NK cells [115]. Strategies targeting activating receptors in addition to CD16 have the potential to stabilize the immunological synapse and lower the threshold of CD16 signaling.

### 4.2. Engager Molecules in Clinical Development

Multi-engager complexes targeting immune cells that are in clinical development have been discussed in other recent reviews; below is an overview of those utilizing CD16 [116]. AFM13, developed by Affimed GmbH in partnership with MD Anderson Cancer Center, is a CD30/CD16 ICE designed for CD30^+^ lymphomas, including Hodgkin lymphoma and peripheral T-cell lymphoma [117]. As a bispecific antibody, AFM13 demonstrated modest single-agent activity in relapsed/refractory Hodgkin lymphoma [117,118]. However, pre-loading allogeneic, cytokine-expanded NK cells with AFM13 ex vivo dramatically increased anti-tumor activity [119]. The Phase 1 clinical trial of this AFM13-NK cell combination (NCT04074746) enrolled 42 patients with CD30+ lymphomas refractory to multiple therapies, a population with very poor outcomes on conventional therapies [120]. NK cells were derived from donor cord blood, cytokine-preactivated, expanded, and then pre-complexed with AFM13 prior to infusion [120]. Results showed the overall response rate was 92.9% and the complete response rate was 66.7% [120]. Importantly, no CRS, ICANS, or GvHD was observed, consistent with the known favorable safety profile of NK cell therapies [120]. At a median follow-up of 20 months, 11 patients remained in complete remission [120]. These results represent some of the most compelling clinical data for any NK cell engager strategy to date.

AFM24 is an EGFR/CD16 ICE that targets EGFR-expressing solid tumors, representing Affimed’s extension of the ICE platform into the solid tumor setting [111]. Preclinical studies demonstrated robust AFM24-mediated NK cell activation and killing of EGFR^+^ tumor cell lines, including cell lines with KRAS or other oncogenic mutations that confer resistance to anti-EGFR antibodies such as cetuximab or panitumumab [111]. The first-in-human Phase I/IIa dose-escalation study of AFM24 monotherapy enrolled 35 patients with advanced EGFR-expressing solid tumors across seven dose cohorts (14–720 mg) [121]. AFM24 demonstrated a favorable safety profile, with toxicities limited to manageable infusion-related reactions, including a single reported case of a grade 3 dose-limiting toxicity [121]. Tumor biopsy data demonstrated activation of innate and adaptive immune responses within the tumor, providing a mechanistic rationale for combination strategies with checkpoint inhibitors or adoptive NK cell therapies [121].

The third Affimed ICE is AFM28, which uses an anti-CD16 effector arm with a CD123-targeting scFv (CD123/CD16) to kill stem and progenitor acute myeloid leukemia (AML) and myelodysplastic syndrome (MDS) cells [113]. In a phase I dose-escalation study (NCT05817058), AFM28 is being evaluated as a monotherapy in patients with relapsed or refractory AML and high-risk MDS [113]. Early clinical data presented in late 2024 and early 2025 demonstrate that AFM28 possesses a manageable safety profile with no dose-limiting toxicities observed up to 300 mg [113]. While two cases of CRS occurred, both were low-grade and resolved without the need for tocilizumab (anti-IL-6R) or other anti-cytokine interventions, and no ICANS was reported [113]. Most notably, in the higher-dose cohorts, AFM28 monotherapy achieved a 40% complete remission [113].

The TriKE platform was pioneered by laboratories at the University of Minnesota in partnership with GT Biopharma (Figure 1A) [101]. The first-generation TriKE, designated GTB-3550 incorporated an IL-15 functional domain between anti-CD16 and anti-CD33 scFvs (CD16/IL-15/CD33) to simultaneously crosslink NK cells to CD33^+^ myeloid malignancy targets and provide in situ IL-15 signaling to drive NK cell activation, persistence, and in vivo expansion. Preclinical studies showed that GTB-3550 induced potent NK cell killing of AML and MDS targets while overcoming myeloid-derived suppressor cells in the tumor microenvironment. In clinical trials, GTB-3550 was shown to be safe and to reduce bone marrow blast levels by more than 60% in AML patients (NCT03214666). GTB-3550 was discontinued in favor of a second-generation TriKE design, GTB-3650. This multi-engager complex replaced the CD16-targeting scFv with a higher-affinity anti-CD16 VHH. The improved design preserves CD33-targeting specificity and cytokine stimulation while increasing CD16 binding. GTB-3650 is being evaluated in Phase 1/2 clinical trials in MDS and AML (NCT06594445).

Innate Pharma and Sanofi have co-developed the ANKET platform, an approach that targets both NKp46 and CD16 on NK cells as well as a tumor-associated antigen [114]. An NKp46/CD16A/CD123 ANKET molecule was under investigation in Phase 1/2 clinical trials for AML and blastic plasmacytoid dendritic cell neoplasm until March 2026, when Innate Pharma announced it was ending work on ANKET.

Dragonfly Therapeutics has developed the TriNKET platform, which targets NKG2D and CD16 simultaneously on NK cells alongside a tumor-associated antigen. DF1001 was designed to engage HER2 on breast and other HER2^+^ cancers, such as urothelial carcinoma, esophageal, lung, and colorectal cancers. In a clinical trial, DF1001 showed a positive safety profile, with no dose-limiting toxicities, and the only adverse events reported were low-grade infusion-related reactions and fatigue (NCT04143711) [116,122].

## 5. Strategies to Overcome ADAM17-Mediated CD16 Shedding for NK Engagers

### 5.1. Anti-ADAM17 Blocking Antibodies

The NK cell engagers discussed rely on CD16 expression to mediate anti-tumor cytotoxicity; however, ADAM17 activation limits the potential of these therapies. As mentioned above, ADAM17 induction occurs in response to various stimuli, including CD16 ligation by engager molecules (Figure 1A) [41,99]. Therefore, it is predicted that the efficacy of NK cell engagers will be enhanced by blocking ADAM17 proteolytic function. Various inhibitors of ADAM17 have been described [51]. Early attempts to inhibit ADAM17 pharmacologically with broad-spectrum small-molecule metalloproteinase inhibitors, including hydroxamate-class compounds that target the zinc-binding active site shared by ADAM17, ADAM10, and MMP family members, were hampered by poor selectivity and dose-limiting off-target toxicities [123,124,125]. For instance, broad-spectrum MMP inhibitors that also target ADAMs, such as marimastat, prinomastat, and tanomastat, failed to progress beyond phase III clinical trials due to lack of efficacy and toxicity [44,126]. Even the two ADAM17-targeted inhibitors, BMS-561392 and TMI-005, retained cross-reactive inhibitory activity and were halted in a phase II clinical trial due to similar efficacy and toxicity issues [44]. These experiences highlight the importance of ADAM17-specific and NK cell-directed ADAM17 inhibition strategies, an objective that can be addressed by the multi-engager complexes described in this section.

**Figure 2:**
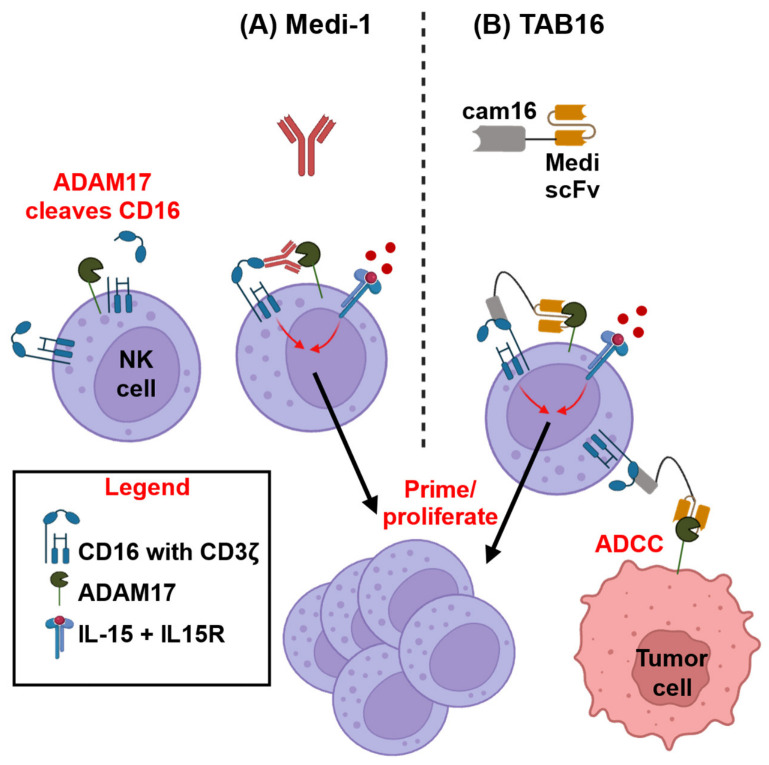
(**A**). ADAM17 cleaves CD16 (left). Medi-1 blocks ADAM17 and its Fc region is engaged by CD16, inducing low-level signaling that synergizes with cytokine stimulation, such as IL-15 (right). (**B**). TAB16: A Medi-scFv/anti-CD16 VHH bispecific engager that simultaneously binds to CD16 in NK cells and blocks ADAM17 function. TAB16 also links CD16 on NK cells to ADAM17 overexpressed on tumor cells to induce ADCC.

MEDI3622 is a specific ADAM17 function-blocking monoclonal antibody developed by MedImmune to block the release of EGFR ligands in solid tumors [127]. Various Fc variants of this mAb have been generated, including the human isotypes IgG1 (Medi-1), IgG4 (Medi-4), Medi-F(ab′)_2_ and Medi-PGLALA to impair FcγR binding, and Medi-scFv [41,42]. Medi-1 blocks ADAM17 function in NK cells, preventing CD16 shedding, while its Fc region is simultaneously engaged by CD16, thereby inducing and prolonging CD16 signaling (Figure 2A) [42]. This synergizes with cytokine stimulation, such as IL-15 and IL-2, enhancing NK cell activation and proliferation [42]. The latter process involves a mechanism of enhanced CD137 (4-1BB) upregulation on NK cells and its engagement of CD137L on other PBMCs [42]. Additionally, blocking CD62L shedding, an ADAM17 substrate that governs NK cell trafficking to proliferative niches, enhanced in vivo NK cell expansion [100]. We are aware of only three other anti-human ADAM17 function-blocking mAbs, D1(A12) [128], which does not have the same functional effects on NK cells as Medi-1 [42], and D8P1C1 and C12, which do not block ADAM17 in hematopoietic cells [129,130].

### 5.2. Next-Generation Engagers Incorporating ADAM17 Inhibition

We developed a novel bispecific engager termed Targeted ADAM17 Blocker CD16 (TAB16), consisting of Medi-scFv to block ADAM17 function linked to a VHH that binds CD16 with high affinity and in a uniform manner (Figure 2B) [41]. TAB16 directed ADAM17 inhibition to NK cells and augmented IL-15-driven activation and proliferation similar to Medi-1 [41]. Importantly, TAB16 also linked NK cells to ADAM17 overexpressed by ovarian cancer cell lines to mediate ADCC, demonstrating dual functionality [41]. The TAB16 engager platform is amenable to modifications, such as the linkage of an IL-15 moiety, which we incorporated into the complex to generate TAB16/15 [41]. By integrating both into a single molecule, TAB16/15 eliminates lot-to-lot variability of the individual components. We show that TAB16/15 maintains NK cell specificity in blocking ADAM17 and in increasing proliferation, while mediating ADCC against ovarian cancer cell lines, with no additional IL-15 required [41]. Our ADAM17-targeting strategy offers a novel means to prevent the shedding of critical receptors in a cell-selective manner.

## 6. Conclusions and Future Directions

NK cell multi-engager complexes represent an important class of immunotherapeutics that address key limitations of monoclonal antibodies and adoptive cell therapies. CD16 is the only NK cell activation receptor capable of inducing full degranulation and cytokine secretion in the absence of co-stimulatory signals [2]. By linking CD16 on NK cells to tumor targets, these engagers can rapidly direct innate immune cell cytotoxicity and circumvent polymorphic differences in CD16 binding affinities for IgG1. However, ADAM17 induction upon NK cell activation by CD16, other activating receptors, various cytokines, and immunosuppressive components of the tumor microenvironment, represents an important impediment to sustained tumor cell killing by ADCC. The anti-ADAM17 mAb Medi-1 blocks ectodomain shedding and promotes cytokine synergy via CD16 signaling, augmenting NK cell priming and proliferative capacity [42]. To address on-target, off-NK cell issues with Medi-1, we have demonstrated that Medi-scFv can be incorporated into anti-CD16 engager complexes for NK cell-directed ADAM17 inhibition [41].

Medi-scFv could serve as a modular component of multi-engager complexes. For instance, Medi-scFv incorporated into TriKEs (i.e., Medi-TriKEs) would block ADAM17 function in NK cells and target them to assorted tumor antigens (Figure 1B). Khaw et al. recently reported a TriKE comprising a VHH to CD16, an IL-15 moiety, and a novel VHH that binds B7-H3, which is overexpressed in a range of solid tumors [105]. Incorporating Medi-scFv into this TriKE may enhance its function in several ways; for instance, by preventing CD16 downregulation in NK cells, resulting in increased ADCC potency and IL-15 synergy. The Medi-scFv and B7-H3-VHH components would enable dual antigen targeting in solid tumors overexpressing ADAM17 and B7-H3, thereby reducing the potential for antigen-escape variants. Medi-scFv could also inhibit the sheddase in tumor cells, retaining NK cell ligands, such as MICA/B and NR3LG1, and preventing the release of EGFR ligands that promote tumor cell growth. Simultaneously blocking ADAM17 on NK cells and tumor cells could optimize the functional transition between activating pathways. As demonstrated by Srpan et al., prolonged CD16 stimulation eventually diminishes perforin expression; however, switching to alternative pathways like NKG2D stimulation can fully restore perforin release [39]. By utilizing Medi-scFv to inhibit ADAM17 on the tumor cell, the crucial NKG2D ligands (MICA/B) are preserved rather than shed. Thus, if NK cells face CD16-mediated perforin decline, they can effectively switch to a sustained, NKG2D-driven cytotoxic response.

Multi-engager complexes containing Medi-scFv are not restricted to enhancing NK cell function and could be used to link other immune cell populations to tumor cells. Bispecific T-cell Engagers and Tri-specific T-cell Engagers (BiTEs and TriTEs, respectively) link cytotoxic T cells to tumor cells for direct killing. The addition of Medi-1 scFv may also benefit these engager complexes. For instance, by (1) blocking key ADAM17 substrates on T cells, such as CD62L, to enhance their trafficking to secondary lymphoid organs and inflammatory tumor microenvironments; (2) blocking ADAM17 substrates on tumor cells; and (3) targeting T cells to tumors that overexpress ADAM17.

Medi-TriKEs may also have broader applications beyond cancer. They could potentially be used to target pathogenic B or T cells involved in autoimmunity or to treat infectious diseases, such as HIV, by including an envelope protein-targeting arm.

The incorporation of ADAM17 inhibition into multi-engager complexes addresses the consequences of the proteolytic checkpoint in NK cells and its role in immune evasion by tumor cells. This approach does more than enhance cytotoxic output by NK cells; it enhances interaction between the effector cell and the malignancy by stabilizing surface ligands and neutralizing pro-tumorigenic shedding. With further clinical development, these Medi-scFv-containing complexes may be pivotal in driving cell-selective ADAM17 inhibition to enhance success in treatments for solid tumors.

## Data Availability

No new data were created or analyzed in this study. Data sharing is not applicable to this article.
